# The Immunomodulatory Drug Glatiramer Acetate is Also an Effective Antimicrobial Agent that Kills Gram-negative Bacteria

**DOI:** 10.1038/s41598-017-15969-3

**Published:** 2017-11-15

**Authors:** Stig Hill Christiansen, Ronan A. Murphy, Kristian Juul-Madsen, Marlene Fredborg, Michael Lykke Hvam, Esben Axelgaard, Sandra M. Skovdal, Rikke Louise Meyer, Uffe B. Skov Sørensen, Arne Möller, Jens Randel Nyengaard, Niels Nørskov-Lauritsen, Mikala Wang, Mihaela Gadjeva, Kenneth A. Howard, Jane C. Davies, Eskild Petersen, Thomas Vorup-Jensen

**Affiliations:** 1 Biophysical Immunology Laboratory, Dept. of Biomedicine, Aarhus University, Aarhus, Denmark; 2 CF and Chronic Lung Infection, National Heart and Lung Institute, Imperial College London, London, United Kingdom; 3 Dept. of Clinical Microbiology, Aarhus University Hospital Skejby, Aarhus, Denmark; 4 Interdisciplinary Nanoscience Center (iNANO), Aarhus University, Aarhus, Denmark; 5 Dept. of Molecular Biology & Genetics, Aarhus University, Aarhus, Denmark; 6 Dept. of Bioscience, Aarhus University, Aarhus, Denmark; 7 Dept. of Structural Biology, Max Planck Institute of Biophysics, Frankfurt, Germany; 8 Core Center for Molecular Morphology, Section for Stereology and Microscopy, Dept. of Clinical Medicine, Aarhus University, Aarhus, Denmark; 9 Center for Stochastic Geometry and Advanced Bioimaging, Aarhus University, Aarhus, Denmark; 10 Dept. of Clinical Medicine, Aarhus University, Aarhus, Denmark; 11 Dept. of Medicine, Division of Infectious Diseases, Brigham and Women’s Hospital, Harvard Medical School, Boston, MA USA; 12 Dept. of Paediatric Respiratory Medicine, Royal Brompton & Harefield Foundation Trust, London, UK; 13 Aarhus University Network for Interdisciplinary Drug Resistance Research, Aarhus, Denmark; 14 Dept. of Infectious Diseases, The Royal Hospital, Muscat, Sultanate of Oman

## Abstract

Classic drug development strategies have failed to meet the urgent clinical needs in treating infections with Gram-negative bacteria. Repurposing drugs can lead to timely availability of new antibiotics, accelerated by existing safety profiles. Glatiramer acetate (GA) is a widely used and safe formulation for treatment of multiple sclerosis. It contains a large diversity of essentially isomeric polypeptides with the cationic and amphiphilic character of many antimicrobial peptides (AMP). Here, we report that GA is antibacterial, targeting Gram-negative organisms with higher activity towards *Pseudomonas aeruginosa* than the naturally-occurring AMP LL-37 in human plasma. As judged from flow cytometric assays, bacterial killing by GA occurred within minutes. Laboratory strains of *Escherichia coli* and *P*. *aeruginosa* were killed by a process of condensing intracellular contents. Efficient killing by GA was also demonstrated in *Acinetobacter baumannii* clinical isolates and approximately 50% of clinical isolates of *P*. *aeruginosa* from chronic airway infection in CF patients. By contrast, the Gram-positive *Staphylococcus aureus* cells appeared to be protected from GA by an increased formation of nm-scale particulates. Our data identify GA as an attractive drug repurposing candidate to treat infections with Gram-negative bacteria.

## Introduction

Multidrug-resistant Gram-negative bacteria are among the most urgent obstacles in the treatment of infectious diseases. Infections caused by extended-spectrum β-lactamases-producing *Enterobacteriaceae* or multidrug-resistant *Pseudomonas aeruginosa* is associated with increased mortality and hospital length of stay^1^. Only a limited number of new suitable antibacterial agents are in development for targeting these infections^2^. Among these, antimicrobial peptides (AMPs) are promising^3^. However, the currently known AMPs are of limited therapeutic value due to systemic toxicity and poor stability^4^. Toxicity has been cited as one reason preventing the clinical use of otherwise promising candidates such as LL-37, a natural AMP generated by proteolysis of human cathelicidin^5,6^, and it explains why the use of other similar compounds are largely restricted to topical applications^4,7^.

The activity of AMPs is often dramatically impeded following contact with the anionic lipopolysaccharides (LPS) in the outer membrane of Gram-negative bacteria^8,9^. Intriguingly, several plants and animals overcome this antimicrobial resistance by deploying multiple peptide isoforms. In this way, temporins and dermaseptins, groups of AMPs isolated from amphibians, provide a specific and fast-acting defence system through synergy between these AMP isoforms^10–14^. Antimicrobial formulations based on such multimerics have previously been proposed^12,14^. However, technical challenges emerge in large-scale, reproducible manufacturing^3^ as well as in the design of safe formulations. One manufacturing option involves the preparation of antimicrobial polypeptides from random polymerization of *N*-carboxy-α-amino anhydride monomers. As early as the late 1950s, it was observed that poly-lysine and random copolymers resembling the natural AMP gramicidin could disrupt bacterial membranes^15^. More recent polymerization techniques enabled the synthesis of binary-block polymers with a well-defined chain composition and length, which adopt the cationic and amphiphilic nature of many AMPs^16^. These copolymers possess high antimicrobial activity^17^. To our knowledge, however, none of the more recently designed antimicrobial copolymers have been tested in humans. Consequently, their toxicity in humans remains unexplored.

The random copolymer glatiramer acetate (GA; also referred to as COP-1)^18^ was among the first effective medical treatments of multiple sclerosis (MS)^19^. From more than 20 years of use in the clinic, GA treatment is known to be safe and well tolerated, even when administered systemically^20–23^. GA copolymers are made from random copolymerization of the acetic anhydrides of l-glutamate, l-lysine, l-alanine, and l-tyrosine in molar ratios that produce polymers with an overall cationic and amphipathic character^24^. It was recently demonstrated that the chemical and biophysical properties of GA are similar to LL-37^25^, including the ability of GA to disrupt lipid bilayers. GA’s composition of random copolymers is reminiscent of temporins and dermaseptins. However, the antimicrobial properties of GA has not been previously addressed.

Here, we report that GA has substantial antibacterial effects. The activity is effective against Gram-negative organisms including *P*. *aeruginosa*, but less effective against the Gram-positive *Staphylococcus aureus*. Clinical strains of *P*. *aeruginosa* from airways of cystic fibrosis (CF) patients displayed varying degrees of susceptibility to GA. Guided by recent progress in characterizing the nanostructure of microbial surfaces^26^ and their interaction with AMPs^27^, we investigated the microbial responses to AMPs in terms of formation of particulate material. Treatment with both LL-37 and GA enhanced formation of nanoparticulates by *S*. *aureus*, whereas Gram-negative species did not form nanoparticulates. Our results suggest that at least some of the challenges in designing safe AMPs using the principles of synergetic AMP isoforms could be overcome by repurposing GA for antimicrobial treatment, particularly against Gram-negative bacteria.

## Results

### The antimicrobial effect of GA on bacterial culture growth

**Figure 1:**
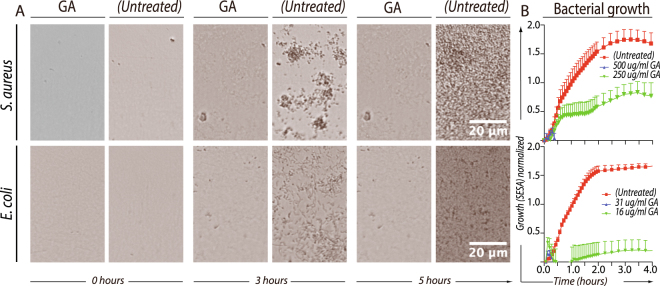
Bacterial growth of *S*. *aureus* and *E*. *coli* assessed by OASTS. Bacteria were incubated with GA to establish MIC from two-fold serial dilutions. (**A**) The oCelloScope™ instrument recorded pictures of the bacterial growth every 10–15 min during the experiment. Pictures from the cultures show the bacterial density at the start (0 h), in the middle (3 h) and at the end (5 h) of the experiments. (**B**) Bacterial growth was measured by the oCelloScope segmentation and extraction of surface area (SESA) algorithm. The applied GA concentrations are indicated in the legend. All data shown represent the mean value and SEM of three independent experiments, each in replicates of two.

The inhibitory of GA on bacterial growth was evaluated by an optical antimicrobial susceptibility testing system (OASTS), which enable real-time cell-by-cell enumeration from time-lapse microscopy. With appropriate adjustments in the initial cell concentration and duration of the experiments to fit the data collection capacity of the instrument, the minimal inhibitory concentration (MIC) of GA was determined for laboratory strains of *S*. *aureus* (Wood 46) and *E*. *coli* (NCTC 10418) with the OASTS (Fig. 1). Representative images are shown of the bacterial density immediately after application of GA (0 h), in the middle of the time lapse (3 h) and at the end of the incubation (5 h) (Fig. 1A). Untreated *E*. *coli* and *S*. *aureus* showed exponential growth to high density within 2–3 h of incubation. With addition of GA to the *E*. *coli* culture, growth was retarded after ∼15 min and with a MIC of 31 µg/ml. This was different from the lower antimicrobial efficacy against *S*. *aureus* with a MIC of 500 µg/ml (Fig. 1B). The OASTS failed analysis of *P*. *aeruginosa* (PAO1) cultures, because the formation of cell aggregates prevented reproducible enumeration by the software (data not shown).

### Fast-acting bactericidal effects of GA and LL-37 on *E. coli* and *P. aeruginosa*

**Figure 2:**
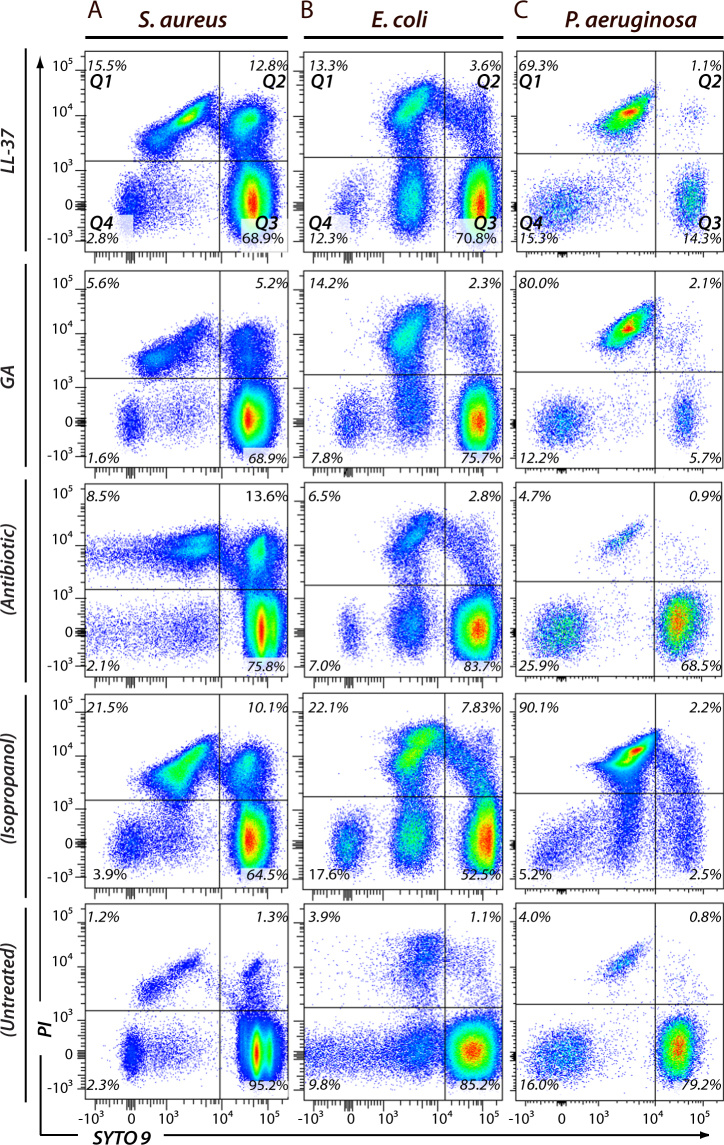
Flow cytometric analysis of *S*. *aureus* (**A**), *E*. *coli* (**B**) and *P*. *aeruginosa* (**C**), exposed to either 50 µg/ml of LL-37, 50 µg/ml of GA, antibiotics or 10% isopropanol. Experiments were carried out in PBS supplemented with 40 mg/ml human serum albumin. Bacteria were incubated with two nucleic acid stains; red-fluorescent PI and green-fluorescent SYTO 9. PI penetrates only bacteria with disrupted membranes. In contrary, SYTO 9 stains all bacteria in a population. From these stains, flow cytometric plots were divided into four quadratic gates (*Q1-Q4*) as indicated with black lines. For each gate, the percentage of total events is stated. Gate Q3 contained viable bacteria, with strong green fluorescence (SYTO 9) and weak red fluorescence intensity (PI). Q1, Q2, and Q4 contained dead bacteria and fragments of bacteria. For antibiotic comparisons, *S*. *aureus* was treated with vancomycin, whereas *E*. *coli* and *P*. *aeruginosa* were exposed to piperacillin/tazobactam. The results shown are representative of a total of four independent experiments.

To further investigate the type of growth retardation exhibited by GA, we used flow cytometry-based assays enabling the immediate assessment of bacterial viability. We investigated the antimicrobial properties of GA and LL-37 on the laboratory strains of *S*. *aureus* (Fig. 2A), *E*. *coli* (Fig. 2B) and *P*. *aeruginosa* (Fig. 2C). With the indications of a fast acting effect, we chose to stain the samples after 30 min of incubation. For comparison, *S*. *aureus* was exposed to vancomycin, while *E*. *coli* and *P*. *aeruginosa* were exposed to piperacillin/tazobactam. Cell killing positive controls were made by isopropanol treatment, with the concentration (10%) adjusted to maintain a population of morphologically intact cells permitting flow cytometric analysis. Cells were stained with propidium iodide (PI), staining only cells with a permabilized, damaged membrane, and SYTO® 9 staining both damaged and undamaged cells. To aid precise counting of cells, a fixed number of microspheres were added to each experiment. By stopping sample inclusion after ∼10,000 counts in the microspheres gate, high comparability was ensured between experiments.

For all bacterial species tested (Fig. 2), the PI/SYTO 9 staining identified major distinct populations of cells in addition to cells with intermediate staining patterns, consistent with other observations using this methodology^28^. Untreated bacteria presented a largely homogenous population of 80–95% of all cells in quadrangle (Q) 3, strongly stained for the green-fluorescent SYTO 9 and only weakly stained by the red-fluorescent PI. All cell-killing agents used in our study, including isopropanol, favoured in general the formation of cell populations highly stained with both PI and SYTO 9 (Q2) or SYTO 9-dim events with a high PI stain (Q1). However, as illustrated by data from a representative experiment (Fig. 2), interesting differences were noted between the treated bacteria and the types of treatment.

For *S*. *aureus*, the combined populations of PI-positive events (Q1 + Q2) reached ∼11% following treatment with 50 µg/ml GA and ∼28% for LL-37 treatment at the same concentration, compared with only 3% in the case of untreated *S*. *aureus* or 32% when treated with isopropanol. This process was driven by increased events in both Q1 and Q2. An increase in a subpopulation of PI-positive events in Q1 with decreased SYTO 9 intensity (<10^3^) followed exposure to both LL-37 and GA (Fig. 2A).

In the case of the Gram-negative species, a 5–8-fold increase in the events in Q1 was observed, with narrowly confined borders in SYTO 9 staining. No change was observed in Q2 (Fig. 2B,C). Among the tested organisms and conditions, the high efficacy of GA against *P*. *aeruginosa* was striking, with the populations in Q1 after GA treatment comprising 80% of all events, exceeding LL-37 at 70%, and reaching levels comparable to isopropanol at 92%. *E*. *coli* was also susceptible to GA treatment, although less so than *P*. *aeruginosa*, with the dead cells reaching a level of 16% following treatment versus 5% without treatment.

**Figure 3:**
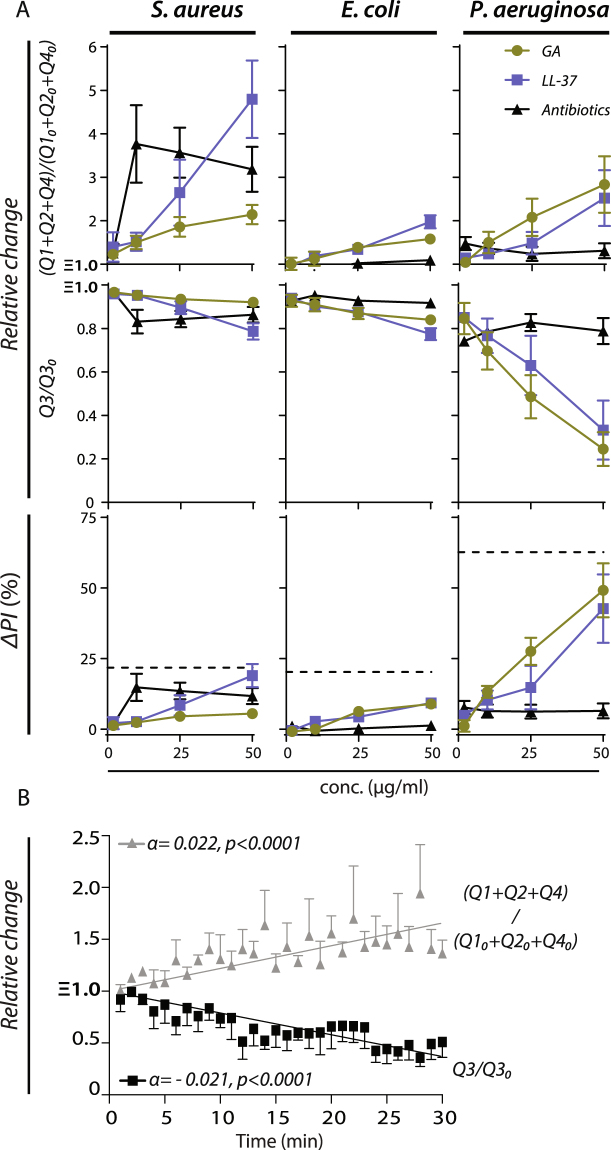
Quantification of antimicrobial activity of GA compared with LL-37 and classic antibiotics using flow cytometry. (**A**) For each species of bacteria (*S*. *aureus*, *E*. *coli*, and *P*. *aeruginosa*) and each type of treatment (LL-37, GA, or antibiotics), the plots show the change in events in Q1 + Q2 + Q4 or Q3 as a function of the applied concentration of antimicrobial relative to untreated bacteria (Q1_0_ + Q2_0_ + Q4_0_ and Q3_0_, respectively). The antimicrobial-induced change in percentage of PI-positive bacterial cells is shown as a function of the concentration applied antimicrobial. For each concentration, the change (ΔPI) was calculated by subtracting the percentage of PI-positive cells in untreated cells (typically less than 5%) from the percentage in the treated cells. Hatched lines indicate the level of PI staining induced by treatment with 10% (v/v) isopropanol. (**B**) Kinetics of bacterial killing. *P*. *aeruginosa* (PAO1) was treated with 50 µg/ml GA in PBS. Bacteria were stained with a mixture of SYTO 9 and PI. Bacterial survival was monitored over 30 min and determined as described in Materials and Methods. Data points were fitted by linear regression. The black line depicts the decrease in viable (Q3) bacteria and the grey line represents the increase of dead bacteria and fragments of dead bacteria (Q1 + Q2 + Q4). All data shown represent the mean value and SEM of four independent experiments.

The relative dose-response levels in cell killing was investigated using the flow cytometry stains and absolute enumeration described above. The increase in PI-positive cells (ΔPI in Q1 + Q2) was measured together with enumeration of the combined events in Q1, Q2, and Q4 versus the events in Q3 enumerating PI-negative/SYTO 9 positive, live cells^28^. To simplify comparison between the independent experiments, results were reported relative to untreated bacteria (Fig. 3). Increasing concentrations of LL-37 and GA resulted in gradually higher PI positivity for all bacterial species, with a particularly noteworthy result for *P*. *aeruginosa*, reaching equivalent levels for LL-37 and GA at 50% using 50 µg/ml (Fig. 3A). The dose-dependency was tested in a Kruskal-Wallis analysis comparing the dosage groups. For *P*. *aeruginosa* groups differed significantly for LL-37 (p < 0.03), GA (p < 0.0001), but not for piperacillin/tazobactam (p < 0.97). The strong PI staining of *P*. *aeruginosa* treated with GA was associated with a three-fold increase in the events in Q1 + Q2 + Q4, differing significantly from the dose-dependent change in events in Q3 at 50 µg/ml GA as analysed by a Mann-Whitney test (p < 0.03). Treatment of *E*. *coli* produced a quantitatively weaker response. However, as for *P*. *aeruginosa*, dosage groups differed significantly in PI staining for treatment with LL-37 (p < 0.0001), GA (p < 0.0005), but not for piperacillin/tazobactam (p < 0.27). The increase in events in Q1 + Q2 + Q4 reached approximately two-fold for treatment with LL-37 (p < 0.03) and GA (p < 0.03), with approximately the same dose-dependency between PI staining and Q1 + Q2 + Q4 events. For *S*. *aureus*, groups differed significantly for treatment with LL-37 (p < 0.01), and vancomycin (p < 0.02), but not for GA (p < 0.15). When using LL-37, *S*. *aureus* produced the weakest response in PI staining among the three bacterial stains, but the largest increase in the Q1 + Q2 + Q4 events at 5-fold using 50 µg/ml LL-37 (p < 0.03). Similarly, for GA treatment, the PI staining of *S*. *aureus* was less than 10%, however, the number of events still increased approximately 3-fold at a concentration of 50 µg/ml (p < 0.03). Unlike the antibiotics treatment of the Gram-negative species, vancomycin treatment of *S*. *aureus* significantly enhanced the PI staining (p < 0.02) and the events in Q1 + Q2 + Q4 compared to Q1 (p < 0.03).

The time-dependent killing by GA were resolved in the flow cytometric assay and showed an almost immediate antibacterial activity upon contact with *P*. *aeruginosa* (PAO1) (Fig. 3B). The increased number of events in Q1 + Q2 + Q4 and corresponding decrease in Q3 showed a statistically significant, linear dependency between time duration and killing, with the same-magnitude rate change (α) also indicating that the assay captured all cells in the experiments.

**Figure 4:**
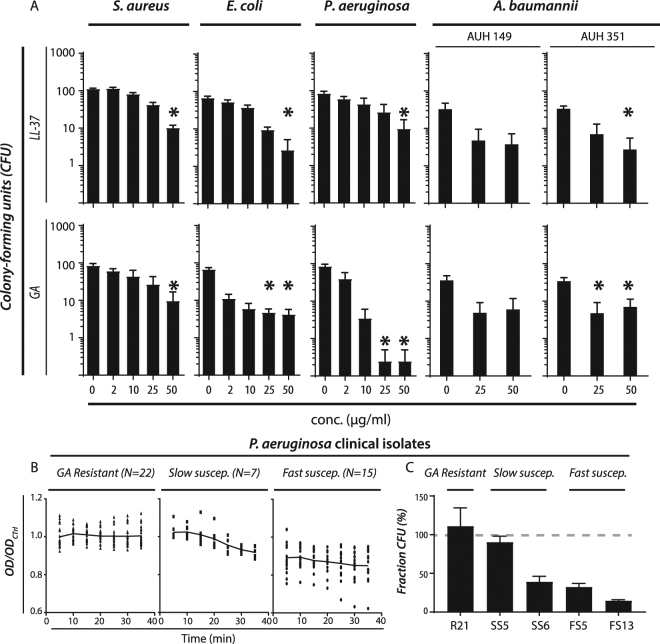
Antimicrobial effects of GA and LL-37 on bacterial laboratory strains and clinical isolates of *A*. *baumannii* and *P*. *aeruginosa* evaluated from colony-forming units (CFU). (**A**) Late-log phase *S*. *aureus*, *E*. *coli*, *P*. *aeruginosa* (PAO1), and two clinical isolates of *A*. *baumanii* were treated with GA or LL-37 for 30 min at 37 °C. After exposure to LL-37 and GA in PBS, bacteria were plated on blood agar plates and the number of CFUs counted. The results shown represent the mean values of four independent experiments with SEM. “*” indicates a statistically significant differences (p < 0.05) from untreated bacteria in a Kruskal-Wallis test with correction for multiple comparisons. (**B**) Analysis of GA’s antimicrobial activity in 44 clinical isolates of *P*. *aeruginosa* from CF patients. Based on raw OD_600_ readings, the isolates were divided into three groups (resistant, slow susceptible, and fast susceptible). For each strain, results are presented as normalized to the final OD_600_ reading after 30 min for untreated control (OD_Ctrl_). (**C**) Analysis of GA’s antimicrobial activity in clinical isolates by CFU counting. One resistant (R21, named according to Supplementary Table 1), two slow susceptible (SS5 & SS6), and two fast susceptible (FS5 & FS13) isolates were plated, either with or without GA treatment as for the experiments quantified by OD_600_. The fraction of CFU from treated bacteria was expressed in % of untreated controls with 100% expressing no change (indicated with a hatched, grey line). For each strain, CFU were counted from 4–10 plates for GA-treated cells as well as for controls. Bars indicate the mean value and error bars SEM.

As an extension on the test on the bactericidal activity of GA and LL-37, the relative number of colony-forming units (CFU) was compared following treatment according to the protocols for the experiments analysed by flow cytometry. To increase the number of Gram-negative species under consideration, *Acinetobacter baumannii* was also included in these analyses, with one multiresistant clinical isolate (AUH 149) and one antibiotic-susceptible isolate (AUH 351). Treatment with GA caused a reduction in as much as 75% of viable Gram-negative bacteria after only 30 min of treatment (Fig. 4A). Both *E*. *coli*, *P*. *aeruginosa*, and *A*. *baumannii* were affected. Among the two *A*. *baumannii* isolates, only the antibiotics susceptible isolate (AUH 351) produced statistical significant results (p < 0.03) with a strong trend for the multiresistant isolate as well (p < 0.06). In *P*. *aeruginosa*, treatment with 25 or 50 µg/ml of GA reduced the CFU almost to the detection limit. From a quantitative perspective, this striking reduction in CFU counts for *P*. *aeruginosa* (Fig. 4A) indicated a more pronounced killing than was found with the flow cytometric assays (Fig. 3A). In general treatment with LL-37 produced weaker responses, ranking the two reagents similarly to the findings with flow cytometry.

A more limited effect was recorded in treatments of *S*. *aureus* similar to the flow cytometry results. Neither LL-37 nor GA were able to strikingly reduce colony formation, and no dose-response was detectable for GA treatment of *S*. *aureus* in concentrations ≥10 µg/ml.

Different strains of *P*. *aeruginosa* vary in their susceptibility to AMPs^29,30^. Whether mucoid or not and sensitivity to antibiotics of the isolates were characterized (Suppl. Table 1). We assessed the influence of GA in suspensions of 44 *P*. *aeruginosa* clinical isolates. Tested isolates had a range of resistance profiles from broad sensitivity to representatives from each of the major antibiotic classes, to a variety of multidrug resistance patterns (including one isolate that was colistin resistant). As before, the response to GA was monitored over a 30-min period in phosphate-buffered saline. Responses of the 44 isolates fell consistently into one of three patterns: *i)* fast susceptibility responders, showing a reduction in OD_600_ at first measurement (i.e., ~5 mins after addition of GA), comprising 34% the isolates; *ii)* slower susceptibility responders, with initial OD_600_ similar to controls but exhibiting a decrease during the 30-min monitored period, comprising 16% of isolates, and *iii)* resistant isolates, a group with an apparent lack of response over the entire 30-min period, comprising 50% of the isolates (Fig. 4B). The susceptibility of each individual isolate was consistent upon replicate and repeat testing. Susceptibility was not related to being mucoid or not or antibiotic resistance as probed with χ^2^ tests (*p* = 0.485 and *p* = 0.896, respectively).

To confirm that the OD_600_ changes obtained reflected bacterial killing, representative isolates from apparently GA sensitive and resistant groups were treated as above and serial dilutions, plating and colony counting was performed. Over all, the results confirmed the influence of GA largely as assessed by the OD measurements. For one resistant strain, viability assessed by CFU at 30 mins remained at 111% of the control (R21 in Fig. 4C). For those strains categorised as GA-sensitive, CFU counting revealed that OD may have been underestimating the killing effect. For the fast susceptible strains FS5 and FS13, the viability was 32% and 15% in CFU counting (Fig. 4C) while the OD measurements indicated only a viability loss of 76% and 82%, respectively. Similarly, for the slow susceptible strain SS6, the CFU counting found a viability of 39% (Fig. 4C), while OD measurement suggested a viability of 88%. A similar observation was made in parallel with *P*. *aeruginosa* (PAO1), where CFU counting found a viability of only 1.3%, similar to the data in Fig. 4A, while OD measurements suggested a viability of 72%. For another slow susceptible clinical strain SS5, estimates were similar with the two techniques at 90% for CFU counting (Fig. 4C) and 92% for OD measurement. Assessment at 30 mins, however, was unlikely to be optimal for such a strain.

### GA antimicrobial activity persist in plasma and pre-formed biofilms

Many AMPs lack antimicrobial activity in blood or other biologically relevant liquids^5,31–33^. Moreover, many bacterial species, including *P*. *aeruginosa*, form biofilms as part of infection, which limits the action of AMPs.

**Figure 5:**
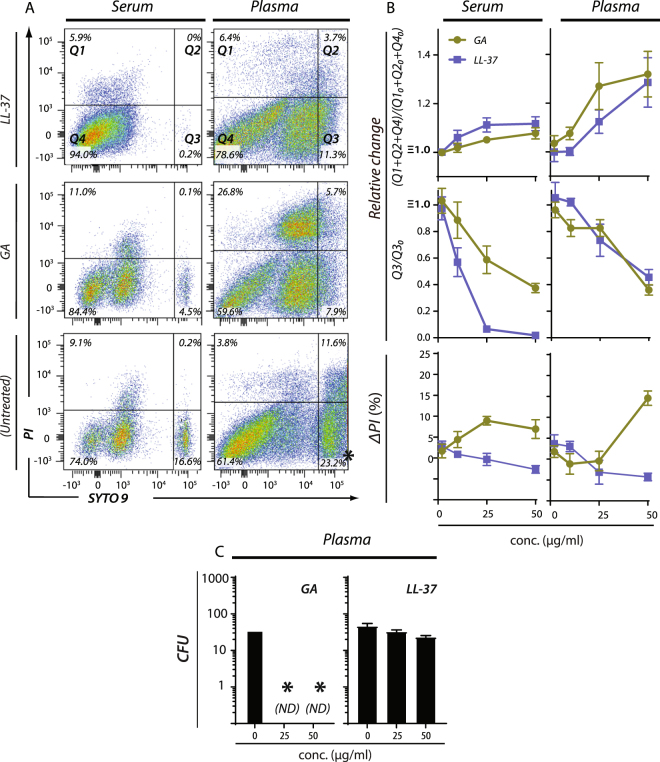
Flow cytometric analysis of antimicrobial activity for GA and LL-37 in human serum and plasma. (**A**) Bacterial isolates of *P*. *aeruginosa* (PAO1) were incubated with varying concentrations of LL-37 or GA or left untreated at 37 °C for 30 min either in plasma or serum. After exposure to GA or LL-37, bacterial cell samples were stained with a mixture of SYTO 9 and PI. (**B**) Plots of the change in events in Q1 + Q2 + Q4 or Q3 as a function of the applied concentration of antimicrobial (2–50 µg/ml) relative to untreated bacteria. The antimicrobial-induced change in percentage of PI-positive bacterial cells is shown as a function of the concentration GA or LL-37. For each concentration, the change (ΔPI) was calculated by subtracting the percentage of PI-positive cells in untreated from treated cells. The results shown represent average values of four independently generated sets of data. SEM are shown as error bars on the curves. (**C**) Antimicrobial effects of GA and LL-37 on *P*. *aeruginosa* (PAO1) in human plasma-containing buffer evaluated by CFU counts. *P*. *aeruginosa* was treated with 25 or 50 µg/ml LL-37 or GA in PBS with 50% (v/v) fresh human plasma, followed by plating and counting as above. (ND, not detected).

We analysed the killing of *P*. *aeruginosa* (PAO1) by GA in human serum and plasma in side-by-side experiments with LL-37, which is known to retain activity under these conditions^34^. Bacterial survival, enumeration and statistical analyses were evaluated by flow cytometry as described above with the incubation time of 30 min (Fig. 3). Unlike experiments in albumin-supplemented buffer (Fig. 3), the number of events in Q4 were relatively high, even without treatment (Fig. 5A), causing relatively moderate changes in Q1 + Q2 + Q4 as part of the treatment with GA or LL-37 (Fig. 5B). Reduction of events in Q3 proved a more robust measure (Fig. 5B), but this came with the emergence of a PI negative/SYTO 9 dim population in Q4, which is difficult to rationalize in terms of viability (Fig. 5A). By contrast, although the PI stain was somewhat attenuated in both serum and plasma, the percentage-positive cells was nevertheless sufficient for concluding that bacterial killing occurred and that GA worked much better than LL-37 (Fig. 5B). To validate these findings further, we also tested the effects by the CFU counting assay used above under conditions with 50% (v/v) human plasma (Fig. 5C). This corroborated the impression from the flow cytometric PI stain, with GA working markedly better than LL-37. Indeed, as noted above, the effect of GA seemed even a stronger antimicrobial agent in this assay than in the flow cytometric analyses.

**Figure 6:**
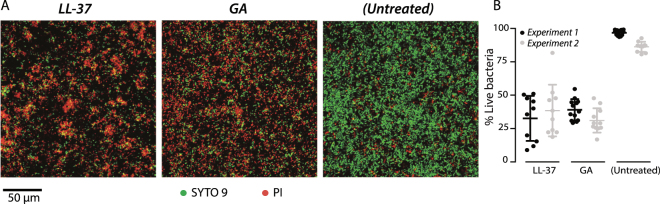
Antimicrobial effects of GA and LL-37 against preformed *P*. *aeruginosa* biofilms. (**A**) LIVE/DEAD staining of *P*. *aeruginosa* (PAO1) biofilms, initially grown for 24 h in IBIDI flow cells and treated for 3 h with 640 µg/ml LL-37 or GA. Living cells are stained with SYTO 9 (green) and dead cells are stained with PI (red). Scale bar 50 µm. (**B**) Quantification of the percentage of live cells in the biofilms after LL-37 or GA treatment or as untreated. Data were from two experiments with each average and standard deviation calculated from 10 images.

The formation of biofilms, such as those associated with chronic infections, may significantly limit the action by antimicrobial agents for example, by trapping cationic agents in neutralizing DNA complexes or maintaining a pH incompatible with antimicrobial function^35^. To test the effect of GA on preformed biofilms, biofilms of *P*. *aeruginosa* (PAO1) were cultivated in flow chambers. LL-37 was included in side-by-side experiments using 640 µg/ml for both compounds, previously determined for LL-37 as the minimal concentration for biofilm eradication in experiments lasting 24 h^36^. After 3 h of incubation, both compounds induced a strong PI staining (Fig. 6A). The average survival of *P*. *aeruginosa* in biofilm exposed to phosphate-buffered saline (PBS), LL-37, or GA for 3 hours was 91%, 34% and 37%, respectively (Fig. 6B). This indicated that the activity of GA was similar to that of LL-37 on preformed *P*. *aeruginosa* biofilms.

### LL-37 and GA induces an envelope stress response in *S. aureus*

The flow cytometric characterization of cellular responses to GA and LL-37 suggested that formation of smaller fragments played a part in the contact with the bacterial cells, especially in the case of *S*. *aureus*. To gain further insight into the nature of these processes through better size resolution than provided by flow cytometry, we quantified the responses by use of nanoparticle tracking analysis (NTA) and transmission electron microscopy (TEM).

**Figure 7:**
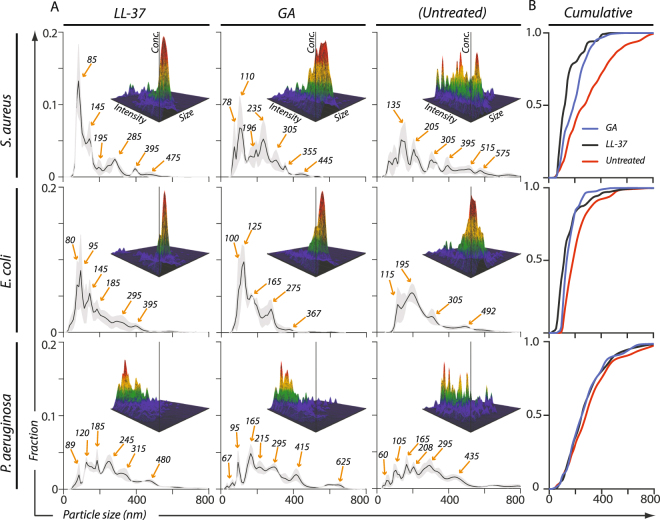
Size distribution of nanoparticulate material in supernatant from *S*. *aureus*, *E*. *coli* and *P*. *aeruginosa* exposed to 50 µg/ml of LL-37 or GA. (**A**) Major debris was cleared from the bacterial supernatants following exposure to either LL-37 or GA or in untreated cultures as described in the Materials and Methods section. The sizes (diameters) of the nanoparticulate material in the nine types of supernatants were quantified by NTA using the NanoSight™ instrument. To facilitate a direct comparison between the size distribution profiles, for each supernatant the concentration of each size of particle was normalized to the total concentration of particles. The normalized concentration of particles was indicated with a solid, black line representing a mean value from three independent experiments, surrounded by a grey area to indicate standard error of the mean. Orange arrows indicate predominant peaks (with sizes in nm). In each panel, three-dimensional graphs show the raw data collected (size vs. intensity vs. concentration) from one representative experiment. (**B**) To further enable comparison between the treatments, the size profiles from the three experiments for each bacterial species were presented in a cumulative plot, each curve representing treatments with GA (blue line), LL-37 (black line) or no treatment (red line).

We recently demonstrated that NTA measurement enables a high sensitivity in measuring damage to cell membranes^25^. Moreover, this technique also measures the size of particles into the nm-regimen, enabling ultrastructural characterization of AMP-induced fragmentation of bacteria. To our knowledge, this approach has not previously been applied in analysis of bacterial fragment formation. NTA was conducted on supernatants from suspensions of the laboratory strains of *S*. *aureus*, *E*. *coli*, and *P*. *aeruginosa* following exposure to either LL-37 or GA and compared with that of untreated bacteria (Fig. 7). For all conditions and species, the particulate material varied in size between ∼80–500 nm. Both in the untreated condition as well as following LL-37 and GA exposure, *S*. *aureus*-derived supernatants presented pronounced spikes in the particle distribution. By contrast, supernatants from the Gram-negative species had a smoother profile (Fig. 7A). In *S*. *aureus*, LL-37 or GA treatment provoked a higher abundance of particles with a size of ∼90 nm. The change in size distribution towards a smaller size of the particles was clear from comparison in a cumulative plot (Fig. 7B). The cumulative distributions were compared in a Kolmogorov-Smirnov test, where the LL-37 and GA treated samples differed significantly from the untreated sample (p < 0.0001 and p < 0.0006, respectively). A similar phenomenon was discernible in the analysis of *E*. *coli* (Fig. 7A,B) for GA treatment (p < 0.002), but not for LL-37 treatment (p < 0.30). The size distribution of particles produced by *P*. *aeruginosa* was essentially unaffected by LL-37 or GA treatment (Fig. 7A,B) although a small significant difference was found between GA treated and untreated samples was found (p < 0.01), but not for the LL-37 treatment (p < 0.08).

**Figure 8:**
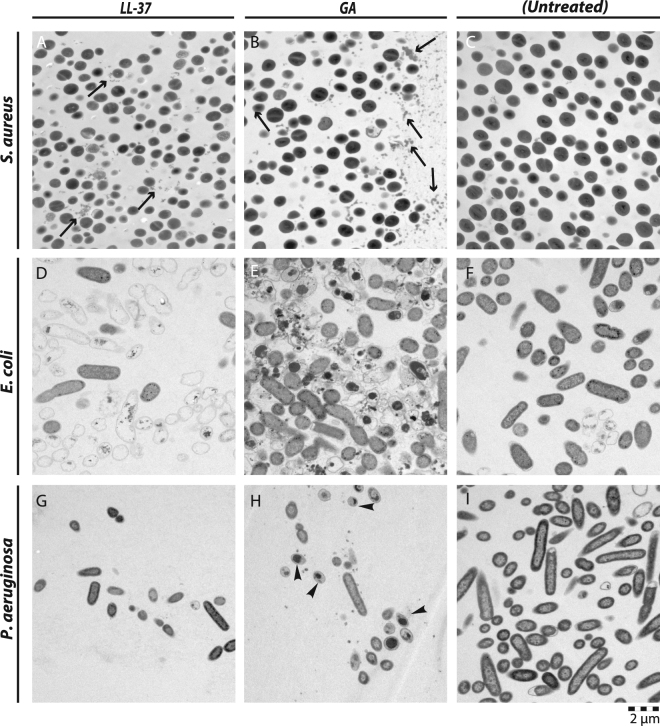
TEM images of *S*. *aureus* (**A**–**C**), *E*. *coli* (**D**–**F**), and *P*. *aeruginosa* (**G**–**I**) exposed to LL-37 (**A**,**D**,**G**), GA (**B**,**E**,**H**), or left as untreated (**C**,**F**,**I**). In the images of *S*. *aureus*, black arrows show debris or microvesicles following treatment with either LL-37 or GA. In the image of *P*. *aeruginosa* after treatment with GA, the arrow heads highlight cells with a condensate similar to those seen with GA-treated *E*. *coli*. Images are representative of two independent experiments, sampling on average 10 images per condition and species in each experiment. Scale bar, 2 µm.

Closely adhering to the conditions used above, TEM imaging was made of S. aurueus following treatment with either LL-37 (Fig. 8A & S1A) or GA (Fig. 8B & S1B). These bacteria appeared largely undamaged from comparison with untreated cells (Fig. 8C & S1C), consistent with the lack of killing of this bacterium observed above. Occasional burst of the cells was observed in treatment with GA (Fig. S1B), but too in frequent to assign this as an effect of GA (Fig. 8B). On the other hand, agreeing with the observations made with NTA, a large number of smaller particulates were also observable following LL-37 or GA treatment (Fig. 8A,B). These structures appeared after exposure to LL-37 and GA and had a size of 50–150 nm.

Untreated *E*. *coli* and *P*. *aeruginosa* demonstrated regularly shaped peripheries surrounding an electron-absorbing cytoplasma (Fig. 8F,I & S1F,I). In *E*. *coli*, LL-37 and GA the treatments produced emptied cellular shapes with a well-defined circumference, more irregular waved than for untreated controls (Fig. 8F & S1F), but clearly coherent (Fig. 8D,E & S1D,E). One of the most distinctive observations was the generation of ghost-like *E*. *coli* cells, almost devoid of cytoplasmic content following LL-37 treatment, with small, electron-dense accumulations (Fig. 8D &S1D). GA treatment produced a more crumbled cellular appearance: in approximately 30% of the cases, large, dense aggregates appeared inside cells without other cytoplasmic content (Fig. 8E & S1E). For treatment of *P*. *aeruginosa*, the total numbers of intact, damaged cells were reduced (Fig. 8G,H) compared with untreated *P*. *aeruginosa* controls (Fig. 8I). As in the case of *E*. *coli*, cytoplasmic aggregates were pronounced following GA treatment, although the size of the aggregates in *P*. *aeruginosa* appeared smaller than those seen in *E*. *coli* cells (Fig. 8H & Fig. S1G–I). Although the influence of LL-37 and GA was pronounced on *E*. *coli* and *P*. *aeruginosa*, remarkably little formation of particulates were observed akin to those found with *S*. *aureus*.

## Discussion

The current lengthy and costly process of *de novo* drug discovery is described as ill-equipped to combat the rapidly emerging drug-resistant Gram-negative pathogens^37^. From this perspective, drug repurposing has been investigated through high-throughput screens as a potential source of antibacterial agents^38^ with notable successes in creating drug leads for treatment of infections caused by amoeba^39^. Known safety characteristics of repurposed drugs often enables swifter testing in patients than in conventional drug development^40^. Typically, auxiliary safety testing in animals is required, while involvement of animal models of disease in appropriate cases can be omitted. This is a particular noteworthy aspect in the case of infectious diseases, where accurate preclinical models of human bacterial infection are notoriously difficult to establish and, by nature, embody significant limitations in terms of gaining insights into drug action against human disease^41^.

In treatment of MS, GA is administered by subcutaneous injection at levels as much as 40 mg three times a week, or 20 mg once daily. The major side effect is erythema, reported in 80% of studied cases, and, in 15–20% of cases, patients experience effects such as chest tightness and shortness of breath. Also supporting the safety of GA, it is the preferred drug choice for pregnant MS patients^20^. Taken together, the use of GA as an antimicrobial to treat potentially lethal infections would not be compromised by its known safety profile. With the already high dosages of GA employed clinically, it seems likely that critical levels of compound to kill microbes can be reached without compromising safety. For example, this could be relevant in treatment by inhalation of lung infections with *P*. *aeruginosa*. The total dosage of 20 mg used routinely in MS therapy would replenish 400 ml of lung fluid to the maximum 50 µg/ml concentration used in our studies. The volume of the epithelial lining fluid in the lower respiratory tract, often a site of *P*. *aeruginosa* infection in cystic fibrosis (CF) patients, has been estimated at 15–70 ml^42^; therefore, infection in this location would only require a dose of 3.5 mg GA, and hence permit a considerable overload of drug delivered to other sites of infection and compensate for losses to zones outside infection foci.

Among *S*. *aureus*, *E*. *coli*, *P*. *aeruginosa* (PAO1), and *A*. *baumannii*, especially the Gram-negative species were susceptible to GA, with *P*. *aeruginosa* the most susceptible in buffer emulating human plasma with regard to salt and HSA concentrations. GA also showed activity against *P*. *aeruginosa* in undiluted human serum and plasma. Here, further validation by use of CFU counting confirmed that human plasma does not interfere with this antimicrobial activity of GA. A barrier commonly found to the effective antibiotics treatment is the embedding of the bacteria in biofilms. However, GA killed *P*. *aeruginosa* in preformed biofilms as efficiently as LL-37, adding yet another valuable property to the antimicrobial activity of GA.

Bacterial fragments, typically in the form of liposomal vesicles, are shed both as virulence factors and as a protective mechanism against various stress signals, including AMPs^43–45^. Using NTA technology, we found that *S*. *aureus* produces particulate material, with sizes around 100 nm, in response to LL-37 and GA treatment. These vesicles were not quantitatively generated by *E*. *coli* or *P*. *aeruginosa*. Formation of this material and greater survival of *S*. *aureus* compared with the Gram-negative organisms encourages the speculation that *S*. *aureus* actively uses the particulates as decoys to protect against otherwise toxic cationic polymers. It is already known that *S*. *aureus* produces cell wall material released to the ambience, likely to be involved in avoiding attack by other parts of the innate immune system^46^. Such nano-particulate material easily enhances the available contacts for AMP binding, consistent with a function as decoy target. From insights on the microbial cell wall structure at the nm scale^47^, notably its assembly in repeating structural units^48^, it is possible AMP-mediated aggregation of these units are part of making the distinct sizes of the released material observe in our study.

Regarding the actual mechanism of killing, inspection of TEM images of the Gram-negative species, particularly *E*. *coli*, revealed an abundance of cellular ghosts, apparently emptied of cytosolic content, or with electron-dense, intracellular condensates, in GA- and LL-37-treated cells to lesser extent. Other AMPs also create cellular ghosts when targeting Gram-negative bacteria^49^. With the established ability of LL-37 to induce nucleic acid complex formation^50^, it seems reasonable to suggest that GA and LL-37 at least partially exert their toxicity by such a process once inside their bacterial targets. This may explain one persistent observation made in our study, namely the apparent stronger antimicrobial effect of GA in the CFU counting assay compared to the measures generated in flow cytometry. GA toxicity through formation of intracellular condensates requires transport of the compound inside the bacteria, which, on the other hand, may not depend on an initial fragmentation or permeabilization of the cellular membranes. In this way, cell fragmentation or PI-staining of permeabilized cells may not fully capture the number of dead bacteria with these compounds.

In CF patients, *P*. *aeruginosa* infection occurs early in life, but can be eradicated in its early stages; however, registry data show that around 60% of adults with the disease are chronically infected^51^. These isolates exist in biofilms, often adopting a mucoid phenotype, adding to a heterogeneous outcome in antibiotics treatment, which was the reason for expanding our observations with the PAO1 strain. Indeed, GA killing was observed in 50% of isolates. One explanation for this proportion seems that the OD-based methodology utilised, suitable for high-throughput testing unlike CFU counting, may have under-estimated the killing effect as also noted for the flow cytometric assays. As another explanation, CF isolates have significant, but broadly differing, sensitivity to AMPs such as cathelicidins^29,30^, which may relate to well-described changes in surface structures such as LPS or to possession of temporate phage; Pf1 was shown in isolates of Liverpool Epidemic Strain to protect against killing by LL-37 by forming inactivating aggregates^30^.

Given the observed fast killing of GA, further drug development work may prove that GA’s best use is as a synergistic treatment alongside conventional antibiotics. Treatment regiments including GA and conventional antibiotics could help ensure that the targeted killing effect occurs, and allow physicians to reduce antibiotic dose, whilst maintaining efficacy thereby reducing the debilitating side effects of some current treatments. Taken together, repurposing existing drugs greatly widens the possibilities of bringing fundamentally novel antibiotics into clinical use within a time frame that can meet the rapidly growing need for treatments of otherwise difficult-to-treat infections with Gram-negative organisms.

## Materials and Methods

### Bacterial strains and culture conditions

Laboratory strains of *E*. *coli* (strain NCTC 10418), *P*. *aeruginosa* (PAO1, ATCC 15692), and *S*. *aureus* (Wood 46, NCTC 7121) were grown on 5% (v/v) blood agar (The State Serum Institute, Copenhagen, Denmark) at 38.5 °C for 16 h in a CO_2_ incubator. Single colonies from the blood agar streaks were transferred to four test tubes containing approximately 10 ml of Todd Hewitt Broth (THB; Sigma-Aldrich, St. Louis, MO). The cultures were incubated with agitation at 37 °C for 16 h and grown to late-log phase. THB is especially used for growth of streptococci; however, we find this rich medium useful for investigations of many other kinds of bacteria due to its low content of undigested proteins.

Clinical isolates of *A*. *baumannii* were obtained from Dept. of Clinical Microbiology, Aarhus University Hospital. One multi resistant isolate (AUH 149) produced the OXA-23 β-lactamase and carried, in addition, the resistance genes *blaADC-25-like*, *blaOXA-64*, *blaPER-1*, *blaTEM-1B*, *aac(3)-IIa*, *strA*, *strB*. AUH 351 is a clinical isolate obtained from blood culture. The isolate is susceptible to meropenem, gentamicin and ciprofloxacin, and has a low MIC to piperacillin/tazobactam.

Clinical *P*. *aeruginosa* isolates from airway secretions (cough swab, sputum or bronchoalveolar lavage) of patients with CF at Royal Brompton Hospital CF Centre are routinely stored on beads (Microbank™, Pro-Lab Diagnostics, Bromborough, UK) at −80 °C. We selected 44 isolates to represent a range of antibiotic-susceptible and -resistant isolates, defined according to standard clinical protocols (Suppl. Table 1). Beads were streaked onto cetrimide agar (Sigma-Aldrich) and grown overnight at 37 °C. Single colonies were inoculated into 10 ml Luria-Bertani broth (LB broth, Sigma-Aldrich) and grown for 14–16 h at 37 °C with agitation at 200 rpm.

### Susceptibility to GA antimicrobial effects tested by OASTS

*E*. *coli* and *S*. *aureus* laboratory strains were grown overnight in Sensititre® cation-adjusted Mueller-Hinton broth with *N*-tris(hydroxymethyl)methyl-2-aminoethanesulfonic acid buffer (CAMHBT, TREK Diagnostic System, Thermo Fisher Scientific, Oakwood village, OH) at 37 °C. Bacterial cell suspensions were adjusted to 0.5 McFarland standard (∼1.5 × 10^8^ cells/ml) using a nephelometer (Sensititre™, Thermo Fisher). Subsequently, bacteria were diluted in CAMHBT to a final inoculum of 5 × 10^5^ cells/ml^52^. Growth was analysed in Nunc Edge™ 96-well plates (Thermo Fisher) by OASTS using the oCelloScope™ instrument (Philips BioCell A/S, Allerød, Denmark). OASTS is a digital time-lapse microscopy technology that scans through a fluid sample, generating time-resolved series of images^53^. Each well was scanned repeatedly every 15 min for the first 2 h and every 10 min for the following 5 h. The OASTS instrument was placed inside an Innova 44 incubator (Eppendorf, Hamburg, Germany) for precise temperature regulation. Digital analyses were made by a custom automation script in MATLAB (Version: 8.0.0.783 [R2012b], The MathWorks Inc., Natick, MA) and image processing was conducted with ImageJ 1.48 v (National Institutes of Health, Bethesda, MD). Growth kinetics was determined by image stack processing based on the contrast-based segmentation and extraction of surface area (SESA). Determination of MIC values for all experiments on quality control reference strains^53^ was carried out in triplicate experiments. Results were reported when at least two out of three MIC results were in agreement.

### Flow cytometric analyses of bacterial viability following incubation with LL-37 and GA

For each bacterial species, 6 ml of late-log phase planktonic bacterial cultures of the *S*. *aureus*, *E*. *coli*, or *P*. *aeruginosa* laboratory strains were harvested by centrifugation at 3096 × *g* for 10 min at 4 °C. The supernatants were discarded and bacteria were resuspended to a final concentration of ~10^8^ cells/ml. From the resuspensions, 1 ml was pelleted and resuspended in 1 ml of PBS (Sigma-Aldrich), supplemented with 40 mg/ml HSA (CSL Behring, Marburg, Germany). Volumes of 975–995 µl of resuspension were treated with 2–50 µg/ml of GA (Copaxone™, Teva Pharmaceuticals, Petah Tikva, Israel), LL-37 (Innovagen, Lund, Sweden), piperacillin/tazobactam (Laboratorio Reig Jofre, Barcelona, Spain), or vancomycin (Xellia Pharmaceuticals, Copenhagen, Denmark) to a final volume of 1 ml. The bacterial suspensions were incubated for 30 minutes at 37 °C with agitation every 15 min. As a control on the viability stains, bacteria were also treated with 10% (v/v) isopropanol (Merck KGaA, Darmstadt, Germany) and incubated for 30 min at 37 °C with agitation. Subsequently, bacteria were pelleted at 3096 × *g* for 10 min at 4 °C and resuspended in PBS. Bacteria were fluorescently stained with LIVE/DEAD™ Baclight^™^ Bacterial Viability and Counting Kit (Thermo Fisher) according to manufacturer’s protocol. Microspheres, included for accurate counting, were vortexed, followed by sonication in a water bath for 5–10 min. A volume of 977 µl of PBS was aliquoted into flow cytometry analysis tubes, plus 1.5 µl of 3.34 mM SYTO 9 and 1.5 µl of 30 mM PI, 10 µl of bacterial suspension, and 10 µl microsphere suspension. Bacteria were analysed in an LSRFortessa™ Flow cytometry instrument (Becton Dickinson, Franklin Lakes, NJ). Each sample was evaluated through ∼10,000 events for the gated microspheres.

For measuring the time rate of killing (kinetics), a late-log phase planktonic culture of the *P*. *aeruginosa* laboratory strain was harvested as above. The supernatants were discarded and bacteria were resuspended in PBS. After resuspension, bacteria were adjusted to ~10^8^ cells/ml. *P*. *aeruginosa* was mixed with SYTO 9, PI, and microspheres and incubated for 15 min at RT. A volume of 237.5 µl of this suspension was aliquoted into wells of a 96-well microtiter plate. To permit fast analysis of the samples, the flow cytometer was started and 12.5 µl GA was then added to the well, bringing the final volume to 250 µl and the GA concentration to 50 µg/ml. Data were collected as above corresponding to a microsphere uptake of 10,000 events accomplished within 1 min.

All flow cytometric analyses, compensation, and gating of flow cytometry data were performed using FlowJo software (Version 10.0.8, Tree Star, Ashland, OR). Single-color controls and an unstained sample were used to locate bacterial populations and to determine compensation settings. The initial gating strategy was based on a gate around the bacterial population on a forward scatter (FSC-A) versus side scatter (SSC-A) plot. The photomultiplier tube (PMT) voltage and the threshold level was adjusted until the entire bacterial population as well as microspheres were on scale on a FSC-A vs. SSC-A plot. Further gating was made by adjusting the green fluorescence channel to ensure that live bacteria stained with SYTO 9 was positioned in the top range of the signal axis. Similarly, PMT voltages of the red fluorescence channel were adjusted to ensure that dead bacteria stained with PI appeared in the top range of the histogram. An unstained sample was applied to confirm that PMT voltages were set appropriately. The viability was depicted by framing bacterial populations in four quadrants in a SYTO 9 versus PI plot as depicted by manufacturer’s instructions. Furthermore, sample matrix was diluted the same as a bacterial sample, confirming that assay background was low and that any background noise on the lower end of the histogram was clearly separated from unstained bacteria.

### CFU counting following exposure to GA and LL-37

Laboratory strains of *E*. *coli*, *P*. *aeruginosa* and *S*. *aureus* as well as the clinical isolates AUH 149 and AUH 351 of *A*. *baumannii* were grown overnight at 37 °C in THB. Bacteria were adjusted to 0.1 McFarland standard (∼3 × 10^7^ cells/ml) in THB and incubated until the bacterial culture reached 0.5 McFarland standard (∼1.5 × 10^8^ cells/ml). Bacteria were pelleted and diluted to a final inoculum of 5 × 10^5^ cells/ml in PBS. Subsequently, bacteria were treated with different concentrations of GA (Teva) or LL-37 and incubated on a shaker for 30 min at 37 °C as described for the flow cytometric experiments above. Two-fold serial dilutions of the incubations were made in 1.5-ml tubes and, for each dilution, a volume of 100 µl was plated on 5% (v/v) horse blood agar plates (The State Serum Institute). The bacterial strains were grown overnight at 37 °C. The resulting CFUs on the blood agar plates were imaged with a digital camera and counted with the aid of the software ImageJ 1.48 v (National Institutes of Health).

### Assessing susceptibility of clinical *P. aeruginosa* isolates to GA

Overnight broth cultures were pelleted at 3900 × *g* for 15 min, supernatant removed, cells resuspended in PBS (Sigma-Aldrich), and the concentration adjusted by to an optical density at λ = 600 nm (OD_600_) of 0.25, which equated to approximately 5 × 10^7^ CFU/ml. To 1.5-ml aliquots of each bacterial suspension, 1.5 μl GA (Biocon, Ltd., Electronic City, Bangalore, India) was added (final GA concentration of 50 µg/mL), samples were vortexed (5 s), and 200 μl was aliquoted immediately into each of 6 wells of a 96-well plate (Thermo Fisher). Control wells contained bacterial suspension with no GA. OD_600_ was measured immediately and at 5 min intervals for 30 mins while plates were shaken at 200 rpm, 37 °C in a FLUOstar Omega plate reader (BMG Labtech, Ortenberg, Germany). We obtained mean OD_600_ values from all 6 wells and normalized them against the strain-specific controls measured after 30 min of incubation. From inspection of developments in the OD_600_ readings, the isolates were grouped into the categories fast susceptible, slow susceptible, and resistant.

CFU counting experiments were done with selected isolates as described in the Results sections. Briefly, isolates were cultivated as for the optical-density based experiments, followed by dilution, plating, and CFU counting essentially as described above for the experiments with the laboratory strains.

### Antimicrobial activity of GA and LL-37 in physiologically relevant environments

Human blood from anonymous healthy volunteers was collected in agreement with the Danish Act on Research Ethics Review of Health Research Projects. The blood was collected in either Vacutainer^™^ EDTA tubes or serum tubes (BD Biosciences). Plasma or serum was recovered by centrifugation. Late-log phase bacterial cultures were harvested, adjusted in either plasma or serum to a final concentration of ∼10^8^ cells/ml, and analyzed by flow cytometry as described above.

For testing of the antimicrobial influence of GA on *P*. *aeruginosa* (PAO1) in human plasma by CFU counting, the assay was conducted as described above. Bacterial turbidity was adjusted to 0.5 McFarland standard in PBS. Subsequently, bacteria were diluted to a final inoculum of 5 × 10^5^ cells/ml in PBS, mixed 1:1 with plasma, and treated with GA (Teva) or LL-37 as already described.

For testing of the antimicrobial activity in biofilms, *P*. *aeruginosa* was grown on tryptic soy agar for 48 hours at 37 °C, and a single colony was used to inoculate LB broth (Sigma-Aldrich, Denmark) for overnight culture at 37 °C and 180 rpm shaking in an Erlenmeyer flask. Two replicate cultures were prepared. Flow chambers (IBIDI µ-Slide VI 0.4, Munich, Germany) were connected to a 1-l media bottle with 37 °C pre-heated LB via a peristaltic pump (Watson-Marlow, Falmouth, UK) as described^54^. The overnight cultures were diluted in fresh LB broth to an OD_600_ of 0.5 (∼ 1.5 × 10^8^ CFU/ml), 0.2 ml was injected into the flow channels for inoculation (3 channels for each biological replicate), and incubated for 30 min at 37 °C with the investigated surface facing up. The flow was then turned on at a rate of 3.5 ml/(h × channel) and the system was incubated 24 h at 37 °C with the surface placed vertically and with an upward flow. After biofilms had formed, the flow was stopped and 0.5 ml phosphate saline buffer (PBS) was gently injected to remove nutrient media from the flow channels. The preformed biofilms were then exposed to 200-µl injections of either 640 µg/ml GA or LL-37 dissolved in ultra-filtered H_2_O or PBS, and then flow cells were incubated under static conditions for 3 h at 37 °C. PBS solutions with SYTO 9 (Thermo Fisher) and PI (Thermo Fisher) were injected and images were captured by confocal microscopy (LSM700; Carl Zeiss AG, Oberkochen, Germany) using 488 nm and 555 nm lasers for excitation. At least 10 representative 2D images were captured along the centre of each channel with a 20 × objective. Finally, ImageJ software (National Institutes of Health) was used for image analyses and quantification of live/dead ratio.

### Nanoparticle tracking analyses and TEM imaging of bacteria

Late-log phase *E*. *coli*, *P*. *aeruginosa*, and *S*. *aureus* laboratory strains were resuspended in PBS and treated with 50 µg/ml of GA or LL-37 and incubated on a shaker for 30 min at 37 °C as described above for the flow cytometry experiments. Subsequently, bacteria were pelleted at 3096 × *g* for 10 min at 4 °C. Supernatants were stored at 4 °C for later analysis. Supernatants were diluted in PBS (Sigma-Aldrich) until the concentration of nanoparticles was acceptable for NTA measurements, approximately 10^8^ particles/ml. NTA measurements were essentially performed as previously described^25^ in a NanoSight™ LM10 system (Malvern Instruments Ltd., Malvern, UK). Camera sensitivity was adjusted to high (i.e., 14–16) and the detection threshold was set close to minimum (i.e., 2–5) to accommodate detection of small particles. The ambient temperature was recorded manually, ranging from 22 to 25 °C.

For TEM analysis, bacteria were cultivated and treated with 50 µg/ml GA or LL-37 as described under section for the flow cytometric analyses. After the treatment, the bacteria were washed three times in PBS. The pellet was submerged in liquefied agar, centrifuged at 3096 × *g*, and allowed to solidify on ice. Fixation, embedding, cutting, and TEM was carried out as described^46^.

### Statistical analyses

For the statistical analysis of the increased number of flow cytometric events in Q1 + Q2 + Q4 and the corresponding decrease in Q3, a Mann-Whitney test was used to test the difference at the maximum concentration applied of antimicrobial agent (50 µg/ml). Differences in PI-staining between groups with different dosage of antimicrobial agent were evaluated by a Kruskal-Wallis test with Dunn’s post hoc to correct for multiple comparisons. The CFU counting results were statistically analyzed using the same procedure further enabling a pairwise comparison between untreated controls and each of the dosage groups. Comparisons between the cumulative particle size distributions were made using the Kolmogorov-Smirnov (K-S) test. A two-tailed p-value below 0.05 was considered significant. All statistical calculations were made with the GraphPad Prism version 6.0 g for Mac OS X, (GraphPad Software, La Jolla, CA).

## Electronic supplementary material

Supplementary Information
