# Supplementary material for: The Immunomodulatory Drug Glatiramer Acetate is Also an Effective Antimicrobial Agent that Kills Gram-negative Bacteria
Source: Sci Rep. 2017 Nov 15;7:15653. doi: 10.1038/s41598-017-15969-3 (PMC5688084; doi:10.1038/s41598-017-15969-3)
Supplement: Supplementary file 1 — Supplementary Information [file 41598_2017_15969_MOESM1_ESM.pdf]

The Immunomodulatory Drug Glatiramer Acetate is Also an Effective Antimicrobial Agent that Kills Gram-negative Bacteria

Stig Hill Christiansen[1], Ronan A. Murphy[2], Kristian Juul-Madsen[1], Marlene Fredborg[3], Michael Lykke Hvam[4,5], Esben Axelgaard[1], Sandra M. Skovdal[4], Rikke Louise Meyer[4,6], Uffe B. Skov Sørensen[1], Arne Möller[7], Jens Randel Nyengaard[8,9], Niels Nørskov-Lauritsen[3,10], Mikala Wang[3,10], Mihaela Gadjeva[11], Kenneth A. Howard[4,5], Jane C. Davies[2,12], Eskild Petersen[10,13,14], Thomas Vorup-Jensen[1,4,13]

[1]Biophysical Immunology Laboratory, Dept. of Biomedicine, Aarhus University, Aarhus, Denmark; [2]CF and Chronic Lung Infection, National Heart and Lung Institute, Imperial College London, London, United Kingdom; [3]Dept. of Clinical Microbiology, Aarhus University Hospital Skejby, Denmark; [4]Interdisciplinary Nanoscience Center (iNANO), Aarhus University, Denmark; [5]Dept. of Molecular Biology & Genetics, Aarhus University, Aarhus, Denmark.[6]Dept. of Bioscience, Aarhus University, Aarhus, Denmark;[7]Dept. of Structural Biology, Max Planck Institute of Biophysics, Frankfurt, Germany; [8]Core Center for Molecular Morphology, Section for Stereology and Microscopy, Dept. of Clinical Medicine, Aarhus University, Aarhus, Denmark; [9]Center for Stochastic Geometry and Advanced Bioimaging, Aarhus University, Denmark; [10]Dept. of Clinical Medicine, Aarhus University, Aarhus, Denmark; [11]Dept. of Medicine, Division of Infectious Diseases, Brigham and Women's Hospital, Harvard Medical School, Boston, MA; [12]Dept. of Paediatric Respiratory Medicine, Royal Brompton & Harefield Foundation Trust, London, UK.; [13]Aarhus University Network for Interdisciplinary Drug Resistance Research.[14]Dept. of Infectious Diseases, The Royal Hospital, Muscat, Sultanate of Oman.

Corresponding author: Prof. Thomas Vorup-Jensen; Biophysical Immunology Laboratory, Dept. of Biomedicine, Aarhus University, DK-8000 Aarhus C, Denmark, phone (+45) 8716 7853; fax (+45) 8619 6128; e-mail: vorup-jensen@biomed.au.dk

Supplementary Table 1: Clinical *P. aeruginosa* isolates data[1]

STRAIN[2]	MUCOID	SAMPLE TYPE	SENSITIVITY TESTING[3]					AGE	GENDER
			Ceftazidime	*Ciprofloxacin*	*Meropenem*	*Tobramycin*	*Colistin*	*years*	
FS1	*Yes*	*Spont. Sputum*	*Sensitive*	*Sensitive*	*Sensitive*	*Resistant*	*Sensitive*	*37*	*Male*
FS2	*No*	*Spont. Sputum*	*Sensitive*	*Sensitive*	*Sensitive*	*Sensitive*	*Sensitive*	*45*	*Male*
FS3	*No*	*Spont. Sputum*	*Resistant*	*Sensitive*	*Resistant*	*Resistant*	*Sensitive*	*25*	*Male*
FS4	*Yes*	*Spont. Sputum*	*Sensitive*	*Resistant*	*Sensitive*	*Resistant*	*Sensitive*	*40*	*Female*
FS5	*No*	*Spont. Sputum*	*Sensitive*	*Sensitive*	*Sensitive*	*Sensitive*	*Sensitive*	*41*	*Male*
FS6	*Yes*	*Spont. Sputum*	*Sensitive*	*Sensitive*	*Sensitive*	*Sensitive*	*Sensitive*	*14*	*Male*
FS7	*No*	*Spont. Sputum*	*Resistant*	*Resistant*	*Sensitive*	*Resistant*	*Resistant*	*46*	*Male*
FS8	*Yes*	*Spont. Sputum*	*Sensitive*	*Sensitive*	*Sensitive*	*Sensitive*	*Sensitive*	*30*	*Male*
FS9	*Yes*	*Spont. Sputum*	*Resistant*	*Resistant*	*Resistant*	*Resistant*	*Sensitive*	*29*	*Female*
FS10	*Yes*	*Spont. Sputum*	*Resistant*	*Sensitive*	*Sensitive*	*Resistant*	*Sensitive*	*36*	*Female*
FS11	*No*	*Spont. Sputum*	*Resistant*	*Sensitive*	*Resistant*	*Sensitive*	*Sensitive*	*26*	*Male*
FS12	*Yes*	*Spont. Sputum*	*Sensitive*	*Resistant*	*Sensitive*	*Sensitive*	*Sensitive*	*38*	*Male*
FS13	*Yes*	*Spont. Sputum*	*Sensitive*	*Sensitive*	*Sensitive*	*Sensitive*	*Sensitive*	*47*	*Male*
FS14	*No*	*Spont. Sputum*	*Sensitive*	*Sensitive*	*Sensitive*	*Sensitive*	*Sensitive*	*38*	*Male*
FS15	*No*	*Spont. Sputum*	*Sensitive*	*Sensitive*	*Sensitive*	*Sensitive*	*Sensitive*	*42*	*Male*
SS1	*Yes*	*Cough Swab*	*Sensitive*	*Sensitive*	*Sensitive*	*Sensitive*	*Sensitive*	*19*	*Female*
SS2	*Yes*	*Spont. Sputum*	*Sensitive*	*Sensitive*	*Sensitive*	*Sensitive*	*Sensitive*	*24*	*Female*
SS3	*No*	*Spont. Sputum*	*Sensitive*	*Resistant*	*Sensitive*	*Resistant*	*Sensitive*	*5*	*Male*
SS4	*Yes*	*Spont. Sputum*	*Resistant*	*Sensitive*	*Resistant*	*Sensitive*	*Sensitive*	*19*	*Female*
SS5	*Yes*	*Spont. Sputum*	*Sensitive*	*Sensitive*	*Sensitive*	*Sensitive*	*Sensitive*	*44*	*Male*
SS6	*Yes*	*Spont. Sputum*	*N/T*	*N/T*	*N/T*	*N/T*	*N/T*	*32*	*Male*
SS7	*No*	*Spont. Sputum*	*Sensitive*	*Sensitive*	*Sensitive*	*Sensitive*	*Sensitive*	*21*	*Female*
R1	*No*	*Spont. Sputum*	*Sensitive*	*Sensitive*	*Sensitive*	*Sensitive*	*Sensitive*	*42*	*Female*
R2	*Yes*	*Spont. Sputum*	*Sensitive*	*Sensitive*	*Sensitive*	*Sensitive*	*Sensitive*	*28*	*Female*
R3	*No*	*Cough Swab*	*Resistant*	*Resistant*	*Resistant*	*Resistant*	*Sensitive*	*13*	*Female*
R4	*No*	*Spont. Sputum*	*Resistant*	*Resistant*	*Resistant*	*Resistant*	*Sensitive*	*22*	*Female*
R5	*No*	*Spont. Sputum*	*Sensitive*	*Sensitive*	*Resistant*	*Sensitive*	*Sensitive*	*48*	*Male*
R6	*Yes*	*Spont. Sputum*	*Sensitive*	*Sensitive*	*Resistant*	*Sensitive*	*Sensitive*	*43*	*Male*
R7	*Yes*	*Spont. Sputum*	*Sensitive*	*Sensitive*	*Sensitive*	*Sensitive*	*Sensitive*	*48*	*Male*
R8	*Yes*	*Spont. Sputum*	*Sensitive*	*Sensitive*	*Sensitive*	*Sensitive*	*Sensitive*	*56*	*Male*
R9	*Yes*	*Spont. Sputum*	*Resistant*	*Resistant*	*Resistant*	*Resistant*	*Sensitive*	*30*	*Male*
R10	*No*	*Spont. Sputum*	*Resistant*	*Resistant*	*Resistant*	*Resistant*	*Sensitive*	*29*	*Female*
R11	*No*	*Spont. Sputum*	*Resistant*	*Resistant*	*Intermediate*	*Resistant*	*Sensitive*	*43*	*Male*
R12	*No*	*Spont. Sputum*	*Sensitive*	*Sensitive*	*Sensitive*	*Sensitive*	*Sensitive*	*31*	*Male*
R13	*Yes*	*Spont. Sputum*	*Resistant*	*Resistant*	*Resistant*	*Resistant*	*Sensitive*	*33*	*Female*
R14	*No*	*Spont. Sputum*	*Sensitive*	*Sensitive*	*Sensitive*	*Sensitive*	*Sensitive*	*16*	*Female*
R15	*Yes*	*Spont. Sputum*	*Sensitive*	*Sensitive*	*Sensitive*	*Sensitive*	*Sensitive*	*25*	*Female*
R16	*Yes*	*Spont. Sputum*	*Sensitive*	*Sensitive*	*Sensitive*	*Sensitive*	*Sensitive*	*24*	*Male*
R17	*No*	*Spont. Sputum*	*Resistant*	*Resistant*	*Resistant*	*Sensitive*	*Sensitive*	*26*	*Male*
R18	*No*	*Spont. Sputum*	*Sensitive*	*Sensitive*	*Intermediate*	*Sensitive*	*Sensitive*	*73*	*Male*
R19	*Yes*	*Spont. Sputum*	*Sensitive*	*Sensitive*	*Sensitive*	*Sensitive*	*Sensitive*	*15*	*Male*
R20	*Yes*	*Spont. Sputum*	*Sensitive*	*Sensitive*	*Sensitive*	*Sensitive*	*Sensitive*	*31*	*Female*
R21	*No*	*Spont. Sputum*	*Sensitive*	*Resistant*	*Sensitive*	*Resistant*	*Sensitive*	*16*	*Female*
R22	*No*	*Spont. Sputum*	*Sensitive*	*Sensitive*	*Resistant*	*Sensitive*	*Sensitive*	*21*	*Female*

[1]Data on clinical isolates of *P. aeruginosa* on mucoidy, sample collection, resistance to classic antibiotics, and age and gender of the patient. [2]Samples were divided into groups of fast susceptible (FS), slow susceptible (SS), and resistant (R) strains. [3]N/T; Not tested.

Fig. S1, Christiansen *et al.*

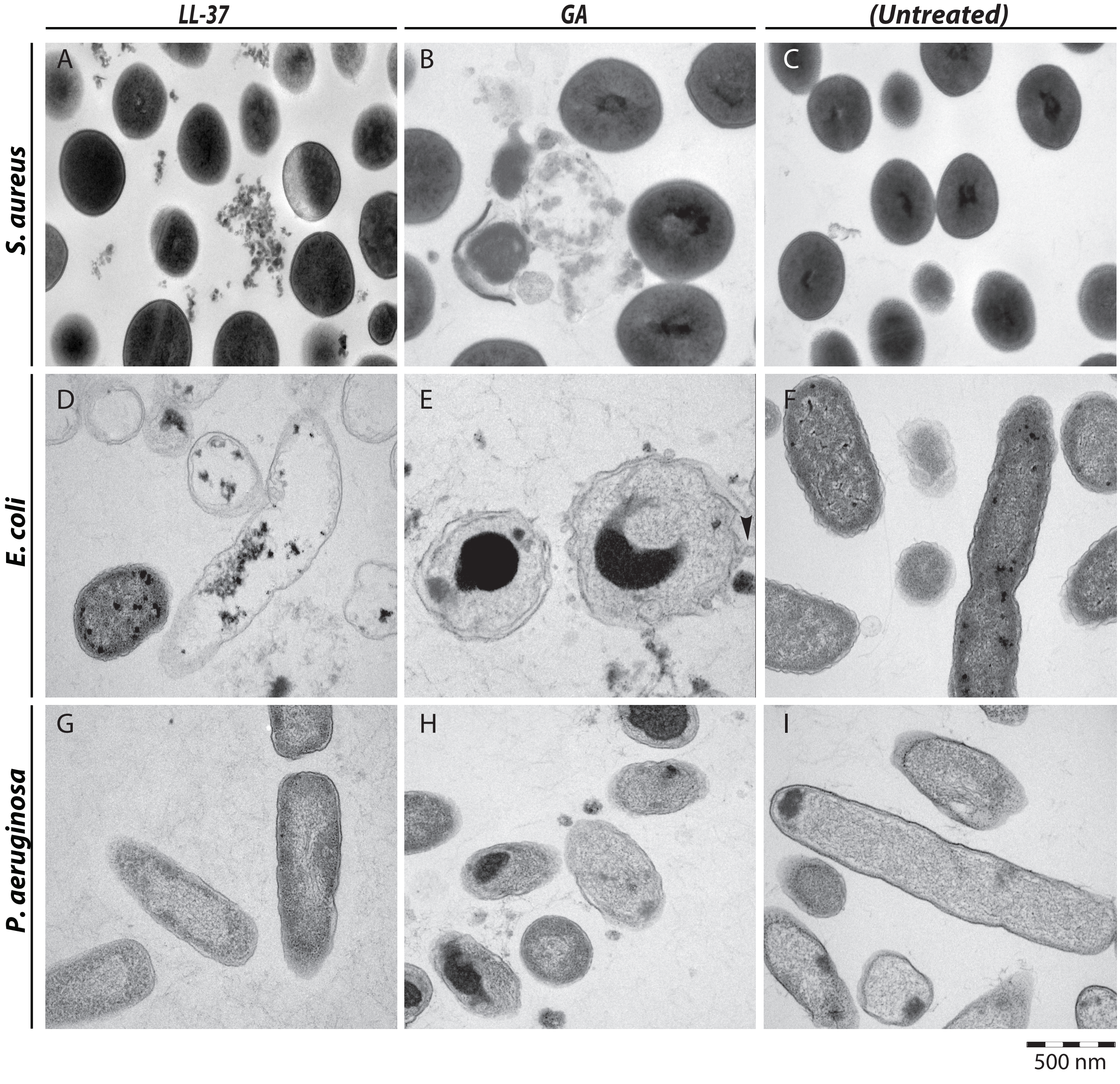

Supplementary Figure 1

TEM images of *S. aureus* (A-C), *E. coli* (D-F), and *P. aeruginosa* (G-I) exposed to LL-37 (A,D,G), GA (B,E,H), or left as untreated (C,F,I). Higher magnification micrographs of the samples shown in Fig. 8. Scale bar, 500 nm**.**
